# Can Drug–Drug Interactions Be Predicted from *In Vitro* Studies?

**DOI:** 10.1100/tsw.2002.144

**Published:** 2002-03-19

**Authors:** Pierre Kremers

**Affiliations:** Advanced Technology Corporation, Institute of Pathology, B23, University of LIEGE, B-4000 SART TILMAN, Belgium

**Keywords:** human, drug, xenobiotic, drug interactions, adverse drug reaction, cytochrome P450, P450, CYP, CYP3A4, drug-metabolising enzymes, inhibition, induction, microsomes, liver, hepatocytes, recombinant enzymes, prediction, extrapolation

## Abstract

Potential drug–drug interactions as well as drug–xenobiotic interactions are a major source of clinical problems, sometimes with dramatic consequences. Investigation of drug–drug interactions during drug development is a major concern for the drug companies while developing new drugs.

Our knowledge of the drug-metabolising enzymes, their mechanism of action, and their regulation has made considerable progress during the last decades. Various efficient *in vitro* approaches have been developed during recent years and powerful computer-based data handling is becoming widely available. All these tools allow us to initiate, early in the development of new chemical entities, large-scale studies on the interactions of drugs with selective cytochrome P-450 (CYP) isozymes, drug receptors, and other cellular entities. Standardisation and validation of these methodological approaches significantly improve the quality of the data generated and the reliability of their interpretation.

The simplicity and the low costs associated with the use of *in vitro* techniques have made them a method of choice to investigate drug–drug interactions. Promising successes have been achieved in the extrapolation of in vitro data to the *in vivo* situation and in the prediction of drug–drug interaction. Nevertheless, linking *in vitro* and *in vivo* studies still remains fraught with difficulties and should be made with great caution.

## DRUG-DRUG INTERACTIONS MAY PRODUCE UNDESIRABLE AND SOMETIMES HARMFUL EFFECTS

The widespread use of drugs has allowed health and comfort of life to improve considerably within our occidental population during the last 50 years and has eliminated several dramatic diseases and controlled or defeated most of them. However, the other side of the coin exists and is called adverse drug effects or reactions (or ADR).

ADR are becoming a growing problem in our hypermedicated societies. The incidence of serious and fatal ADR is very high, affecting more than 6% of the hospitalised patients and causing more than 100,000 deaths per year in the U.S.[1,2]. According to Heerey et al.[3], 7% of acute hospital admissions in Ireland result from ADR, of which 25% are due to drug–drug interactions (hereafter called DDI). ADR arise in 3% of the patients receiving statins, drugs widely prescribed to lower the cholesterol level. Most of these statin-linked ADRs are due to their coprescription with other lipid-lowering agents and are thus the consequence of DDI. ADR results most often from drug abuse, misuse, overprescription, bad prescriptions, wrong comedication, and genetic factors, but also from effects produced by alimentary products, environmental contaminants, etc.[4].

## DRUG-DRUG INTERACTIONS (DDI)

DDI (see Table 1) are one of the sources of ADR. Recent experience has shown that this could lead to medical and financial disaster, including death of badly treated patients and dramatic financial outcomes for the producing company. Cerivastatin (Baycol®/Lipobay®) was withdrawn from the market[5] after the death of more than 30 patients. This caused an immediate 25% loss of value for Bayer shares, due to a reduction in operating profit of about 800 million € for 2001[6,7].

The most common DDI may be understood in terms of metabolic alterations, primarily associated with changes in the activity of cytochrome P450 enzymes. The first and principal consequence is a modification of the drug plasmatic concentration, half life, and clearance. This review will concentrate mainly on DDI involving cytochrome P450 enzymes.

**TABLE 1**
**Some Examples of Drug–Drug Interactions and Their Consequences**

| | |
|---|---|
| Terfenadine and grapefruit juice may produce cardiac arythmia | [4] |
| Rifampicine and contraceptives may produce contraception failure | [8] |
| Rifampicine may cause allograft rejection by inducing cyclosporine metabolism | [60] |
| Triazole antifungals like ketoconazole, administered with cyclosporine, may lead to toxic effects of cyclosporine | [8,9] |
| Statins, cholesterol-lowering drugs, combined with gemfibrozil may lead to fatal rhabdomyolisis | [9] |
| Excessive sedation occurs in children when midazolam is administered with grapefruit juice | [10] |
| Excessive reduction in blood pressure may occur in patients receiving both angiotensin-converting enzyme inhibitors and statins for a long period of time | [11] |
| Increase of saquinavir blood levels by coadministration of ritonavir, a CYP3A4 inhibitor, is a potentially beneficial interaction exploited during HIV treatment | [61] |
| St. John's wort drastically increases the metabolism of several CYP3A4-metabolised drugs like indinavir, cyclosporine, ethinyl estradiol, taxol, delavirdine, digoxin, phenytoin, etc. | [12] |

## DRUG-METABOLISING ENZYMES

The drug-metabolising enzymes are responsible for the metabolism of a large group of substances that are foreign to the normal environment of the cell and are better called xenobiotics.

Xenobiotic-metabolising enzymes constitute a battery of enzymatic proteins devoted to the chemical transformation of lipophilic compounds into water-soluble, inactive products that can be easily eliminated from the cell. They are often classified into three phases:

1. During phase I, enzymes, namely the cytochrome P450–associated enzymes, introduce polar function on their lipophilic substrates.
2. During phase II, enzymes conjugate them with biochemical entities like sulfate, glutathion, glucuronic acid, etc. to transform them into water-soluble entities that can be eliminated from the cell and from the organism, namely via bile or urine.
3. During phase III, transport proteins carry out the excretion of drugs and their metabolites from the cell.

During the last 3 decades, considerable progress has been made in the study of drug metabolism. Most of the drug-metabolising enzymes have been identified, characterised, and sometimes even purified. For most of these enzymes, the DNA coding sequences are known and isolated, allowing heterologous expression of these enzymes in genetically modified cells. Great progress has been achieved in the regulation of these enzymes[13].

Environmental factors (air and water pollutants), food constituents and additives, social habits, and products (alcohol, cigarette, drugs, etc.) are involved both as substrates and regulation factors for these enzymes.

DDI shows up when the efficacy or the toxicity of a drug is modified by the coadministration of another substance. This other substance is not necessarily another drug but may be any other nutrient or chemical of environmental origin[9].

Pharmacokinetic interactions most often result from changes in drug metabolism. Drug metabolism varies considerably from one individual to the next, due to genetic, physiological, pathophysiological, and environmental factors, and to interactions of drugs or drugs and xenobiotics at different sites of action in the organism[8,14].

Several web sites are devoted to the P450 enzymes. A general and well-illustrated introduction to P450 biochemistry is available at http://home.earthlink.net/~cpardee/. A directory on P450-containing systems may be consulted at www.icgeb.trieste.it/~p450srv, while http://drnelson.utmem.edu/CytochromesP450.html is mainly devoted to the nomenclature of the different P450 isozymes. A drug interaction table for clinicians and general practitioners is available at http://medicine.iupui.edu/flockhart. Practical information on drug prescription and possible interactions may be consulted at http://pharmacotherapy.medscape.com. A site by M. Oscarson and M. Ingelman-Sundberg is devoted to the different CYP alleles at http://www.imm.Ki.se/CYPalleles.

## *IN VITRO* APPROACH

Drug–drug as well as drug–xenobiotics interactions occurring during metabolism can be studied using *in vitro* approaches. One drug or xenobiotic may modify the metabolism of another one by various mechanisms: enhancing the metabolism or reducing the metabolism by acting on the expression or on the activity of the responsible enzyme.

Experimental tools are available to study the mechanism of action via *in vitro* approaches. *In vitro* approaches offer several decisive advantages: they allow us to study a large number of products simultaneously in well-defined and reproducible conditions, they are not too expensive, and they are very rapid compared to clinical or animal experimentations.

A general consensus among the scientists from academic, pharmaceutical laboratories, and drug agencies involved in this field confirms that *in vitro* studies are a powerful, effective, and particularly useful tool. Official authorities like the U.S. Food and Drug Administration (FDA) [see on http://www.fda.gov/cder/guidance/index.htm] and the European Agency for the Evaluation of Medicinal Products (EMEA) [see guidance CPMP/EWP/560/095 on http://www.eudra.org/emea.html] strongly recommend preliminary *in vitro* studies on new chemical entities during the development of new drugs.

### How Can Drugs Compete at a Metabolic Level?

A drug may induce the activity of one or several drug-metabolising enzymes and consequently accelerate its own metabolism or that of another drug. On the other hand, some products, namely cytokines, may repress some enzymes. These consequences usually result from an effect on the regulatory elements controlling the expression of the concerned enzyme.

A drug may interact directly at the enzyme level by different mechanisms:

- Competition for the same active site
- Modification of the enzyme kinetics by an allosteric effect or a noncompetitive effect
- Alteration of the enzymatic activity by alteration or degradation of the enzyme (suicide substrates)

### What Kind of Experimental Approach Can Be Used In Vitro to Predict Drug–Drug Interactions?

As far as interactions at the level of xenobiotic-metabolising enzymes are concerned, the approach used depends on the mechanism of interaction. Experimental models used for these studies include the following:

- Microsomes, a subcellular preparation of endoplasmic membranes, the most universally used *in vitro* model (in some circumstances, other subcellular fractions like S9, cytosol, or mitochondria may be used)
- Hepatocytes in culture or liver slices
- Recombinant enzymes resulting from genetically transformed organisms in order to express (or overexpress) a specific enzyme, namely human liver cytochromes

Methodological considerations concerning these experimental models were extensively discussed in a previous paper[14]. Microsomes and recombinant enzymes are principally used to study inhibition and activation effects occurring at the enzyme level.

## INDUCTION OF DRUG-METABOLISING ENZYMES

Cultured cells or tissue slices are used to study induction effects. Functional cells, in which the necessary activators and regulators are expressed, are required in order to reveal an induction effect (a modification in the expression of enzymes). Some cell lines, previously transfected in order to express the necessary enzymes, may also be used for this purpose. To measure the effect of an inducer, it is necessary to pretreat the cells for a few hours before detecting any effect, since the effect is time- and concentration-dependent.

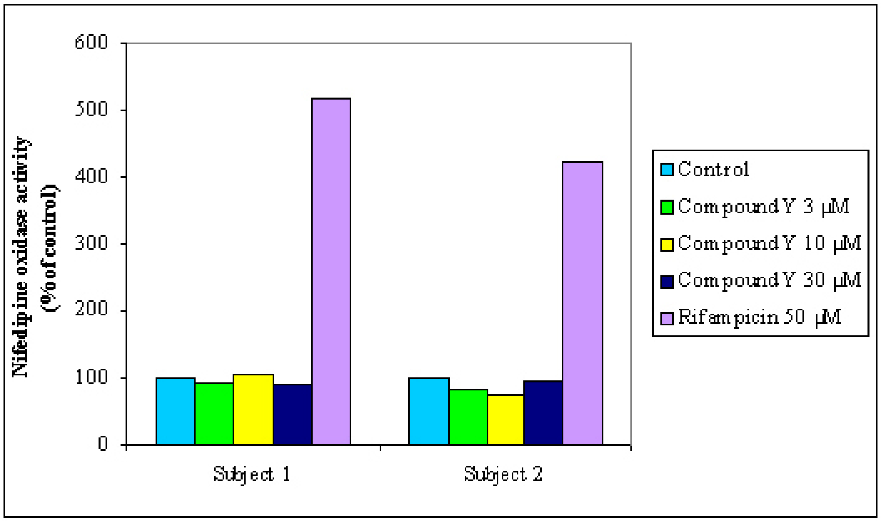

**FIGURE 1.** Induction of CYP3A4 in cultured human hepatocytes by rifampicin, a reference inducer and by a new NCE, called Y, at different concentrations. Figure and data kindly provided by Claire Guyomard, from BIOPREDIC, Rennes (France).

Currently, functional human hepatocytes, hepatic slices, and various cell strains are available from different commercial sources [www.biopredic.com, www.invitrotech.com, www.humanbiologics.com, www.xenotechllc.com]. Their quality, or metabolic capacity, varies as a function of their origin, the isolation and conservation procedure, and the culture conditions used for the experiment. Therefore, results may vary considerably from one laboratory to the next. For this reason it is also imperative to run positive and negative controls within each experimental session to assure a possible comparison and a validation of the process.

Various endpoints can be used to evaluate the extent of induction. They include measurement of enzymatic activities, measurement of the concentration of a specific protein (specifically, via immunological methods), and measurement of mRNA using specific probes[15]. Quantitative RT-PCR using the TaqMan technology can be applied to quantify a CYP mRNA and thus provide a direct measurement of the extent of induction. Necessary tools like cDNA probes and antibodies are now available from various commercial sources.

In Fig. 1, the CYP3A4 activity, nifedipine oxydase, was measured on cultured human hepatocytes exposed to rifampicin, a reference inducer, or to compound Y, a new chemical entity (NCE) suspected to be an inducer. The data clearly indicate that compound Y was unable to induce CYP3A4 in cultured human hepatocytes.

## INHIBITION OF DRUG-METABOLISING ENZYMES

Inhibition of drug metabolism may result from different mechanisms:

- Competition of two or more products for the same enzyme
- Noncompetitive inhibition
- Modification of the enzyme or of the contribution of coenzymes or other enzymes in a biochemical pathway
- Modification of the active site of the enzyme either directly or via metabolisation

Therapeutically important CYP3A4 inhibitors include antifungals like ketoconazole and itraconazole, some antibiotics like clarithromycin and erythromycin, protease inhibitors like ritonavir, anticancer drugs like paclitaxel[16,17], and also natural components of grapefruit juice[9].

**TABLE 2**
**Some Undesirable Side Effects Enhanced by CYP3A4 Inhibitors[9,10,11]**

| Drug | Side Effect |
|---|---|
| Terfenadine, Astemizole, Pimozide, Cisapride | Ventricular arrythmia associated to QT prolongation |
| Some statins | Rhabdomyolysis |
| Midazolam, benzodiazepine | Increased sedation |
| Sildenafil, phosphodiesterase inhibitors | Hypotension |
| Carbamazepine | Ataxia |
| HIV protease inhibitors | Increased bioavailability |
| Angiotensin converting enzyme inhibitors | Reduced blood pressure |
| Cyclosporine | Increased immunosuppressive effect |
| Cyclosporine | Renal toxicity |

When used in combination with drugs metabolised by CYP3A4, these products may considerably modify the plasmatic concentration of these drugs and, consequently, their pharmacological or toxic effect; some examples are given in Table 2. It also happens that the inhibitory effect can be exploited in order to increase a therapeutic effect, while reducing the administered dose, of an expensive medication; this is the case for the tri-therapy in HIV infections.

The mechanism and the extent of the inhibition can be studied on isolated enzymes, namely human liver microsomes, or on material from heterologuous expression. These preparations are also available from different commercial sources [www.biopredic.com, www.gentest.com, www.cypex.co.uk].

## PRELIMINARY IDENTIFICATION OF THE ENZYME

Before starting an inhibition study, it is necessary to first identify the enzyme involved. Most of the reactions are catalysed by cytochrome P450s. These enzymes are hemoproteins, classified in families and subfamilies, called CYPs[18]. For instance, cytochrome P450 CYP3A4 metabolises a large variety of drugs and is also the most abundant CYP in human liver and small intestine, being able to affect presystemic as well as systemic drug metabolism[9].

Several experimental approaches have been developed for the identification of the CYP involved in the metabolism of a drug[19,39]:

- Use of selective and specific substrates in correlation studies
- Use of specific inhibitors
- Use of antibodies specific for each P450 form
- Use of enzymes issued from heterologuous expression to develop cells overexpressing a single CYP

### Preliminary Requirements

It is absolutely necessary to first develop a sensitive and specific enzymatic assay in order to measure the metabolism of the examined drug and to define the corresponding kinetic properties like Km and Vm. Since we are dealing with membrane proteins, the correct terms are *apparent* Km, Vm, or Ki. Once this has been accomplished, it becomes possible to start assays in order to identify the CYP that supports the reaction.

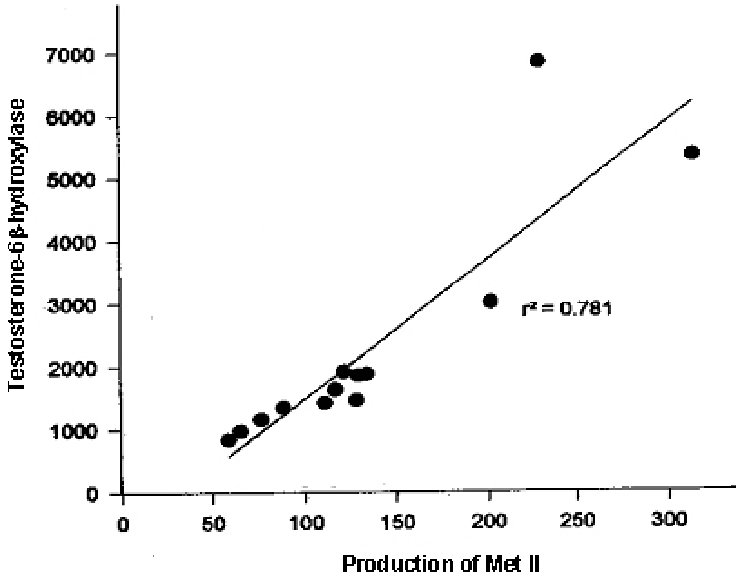

**FIGURE 2.** Correlation between testosterone-6β-hydroxylase activity and the biosynthesis of metabolite 2 of a tested NCE in different human liver microsomes. Unpublished results from P. Kremers, ATC Liège, Belgium.

## Correlation Studies

The enzymatic activity can be correlated with other measures performed on the same enzymatic preparations but using specific substrates. Lists of substrates are available in the literature[14,19] and on some Web sites[20]. Appropriate incubation and measuring conditions must be applied to perform this experiment: substrate in saturating concentration, incubation time and enzyme concentration chosen to assure a linear response, and all other parameters (buffer, cofactors, etc.) optimised. At least ten different enzymatic preparations or microsomes from pooled liver samples should be used in this process.

Fig. 2 shows the correlation between the rate of production of Met II, the principal metabolite of a NCE, and testosterone-6β-hydroxylase activity measured on several human liver microsomal preparations. The positive correlation indicates that MET II is most probably generated by CYP3A4.

## Specific Inhibitors

Inhibitors specific for the different CYPs are available in the literature[19,21,22,23] as well as on the Web[20]. Inhibitors should be used in well-defined conditions to assure a reliable diagnostic[19]. Inhibitors specific for human liver P450s are not necessarily efficient for the inhibition of the corresponding rat liver enzymes and vice versa[24]. To assure a selective and interpretable inhibition, the substrate concentration should be lower than or equal to the Km value and the inhibitor concentration should be similar to the Ki value.

Practically, it is preferable to use at least two inhibitor concentrations in order to control the concentration depending response. Depending on the inhibitor and its mechanism of action, it may or may not be necessary to perform a preincubation of substrate, inhibitor, and enzyme, or of two of these compounds before starting the real incubation. Fig. 3 illustrates typical results of such an inhibition experiment.

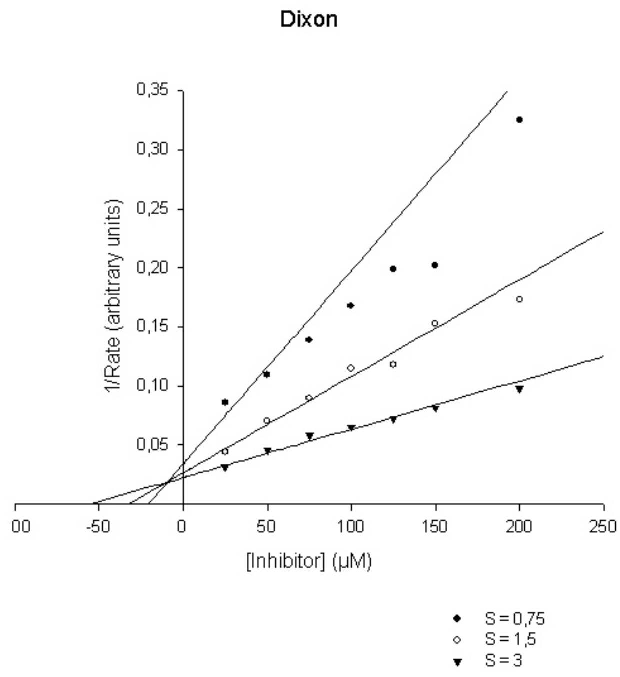

**FIGURE 3.** Dixon plot illustrating the competitive inhibition of CYP3A4 activity by fluoxetine (Ki = 9.5 µM) in human liver microsomes. Unpublished results from P. Kremers, ATC Liège, Belgium.

## Specific Antibodies

Specific antibodies can be used in two ways:

1. Using an immunoassay (ELISA or immunoblot)[25] to measure the amount of protein recognised by the antibody, followed by a correlation with the corresponding or studied enzymatic activity
2. Inhibition of the enzymatic activity by the antibody

It must be noted that not all antibodies are inhibitory, and that the specificity of the antibodies is never perfect. Therefore, this approach has to be used with some caution. To circumvent this drawback, Boobis and collaborators[26] prepared monospecific antibodies against the different human P450s by immunising rabbits with synthetic peptides representing small regions of the P450 amino acid chains.

Table 3 shows an example of such an inhibition study leading to the conclusion that the two measured metabolites of the NCE tested are most probably generated by CYP3A4, but this result in itself is insufficient to definitively identify the enzyme.

Specific polyclonal and monoclonal antibodies are available from different commercial sources (namely Cypex, Gentest, Panvera, and Oxford Biomedical Research).

**TABLE 3**
**Effect of Specific Antibodies on the CYP3A4 Activity and on the Synthesis of NCE Metabolites**

| *Antiserum* | Testosterone-6β-Hydroxylase (nmol/min·mg) | NCE Metabolite I (nmol/min·mg) | NCE Metabolite II (nmol/min·mg) |
|---|---|---|---|
| Control | 6.6 | 1.8 | 2.7 |
| Anti-CYP3A4 | 2.9 | 1.1 | 2.0 |

**TABLE 4**
**Comparison of Human Liver Microsomes and CYP3A4 Expressing Supersomes to Metabolise Testosterone and a NCE**

| Enzymes | Testosterone-6β-Hydroxylase (nmol/min·mg) | Biosynthesis Of Metabolite I (nmol/min·mg) | Biosynthesis Of Metabolite II (nmol/min·mg) |
|---|---|---|---|
| Human liver microsomes | 3.5 | 1.7 | 1.6 |
| CYP3A4 expressing supersomes | 5.4 | 1.2 | 1.0 |

## Heterologously Expressed Enzymes

Different types of cells have been genetically transformed in order to specifically express a particular CYP, namely the human CYPs[27,28]. Microsomes from these cells are currently available from various commercial sources, along with all the technical indications for their optimal use[29]. This is a very powerful tool, allowing exploratory screening experiments as well as systematic search for inhibitory effects and examination of the mechanism of action.

These enzymes can be effectively used as “pseudo purified” enzymes to screen compounds as possible substrates for the corresponding CYP. The principal advantage of these experimental models lies in the fact that they use “isolated” enzymes, and that, consequently, no interference of other isozymes will disturb the analysis or/and complicate the interpretation of the data[23]. Nevertheless, setting up a sensitive assay to measure the produced metabolites remains, of course, a prerequisite. Table 4 shows an example of results obtained during a study devoted to the identification of the CYP involved in the metabolism of the NCE already used in Fig. 2 and in Table 3. Taken separately, these data are insufficient to made a definitive enzyme identification. But together, these different approaches constitute a piece of evidence that allows confident identification of the responsible enzyme.

Recombinant human CYPs are also advantageously used to search for inhibitors and to determine inhibition constants. Ki values obtained were very similar to those determined on human liver microsomes[30].

For a specific enzyme (e.g., CYP3A4), inhibition potential and/or activation potential of several compounds varies in a substrate-depending manner[31], indicating that CYP3A4 inhibition data, at least, should be interpreted with caution.

Nevertheless, these overexpressed enzymes may produce false positive results. The biological environment of these enzymes (lipid membranes, cofactors, and other enzymes like reductase and cytochrome b5 involved in the reaction) may be quite different form the normal biochemical situation in a living cell. In the P450 field, absolute substrate specificity does not exist, and recombinant enzymes may often support reactions that the corresponding enzyme will not perform *in vivo* or even in liver microsomes. The protein and lipid environment of CYPs is relatively different in liver microsomes and in heterologously expressed systems. In microsomes, different CYPs coexist and may interact; their actual specific concentration is not precisely known. In addition, it may be difficult to define optimal reaction conditions for enzymatic preparations derived from heterologously expressed systems and these conditions may be very different from those applied to liver microsomes[14]. Therefore it is necessary, at least in case of low-positive or unclear response, to perform a control on liver microsomes.

Metabolic data obtained from recombinant microorganisms should be considered with caution, since an apparently major biotransformation pathway may be minor *in vivo*, if the abundance of the isozyme in the metabolising organ is low.

In common practice, it appears that a clear and definitive identification of the CYP involved in a particular metabolic reaction requires the combination of at least two of these possible approaches — the most efficient approach being to start with the use of the specific inhibitors, and then to confirm the first indications using heterologously expressed proteins. Specific antibodies are used when available but are seldom inhibitory. Correlation studies are only occasionally used, namely to confirm a particular activity, since they are time- and enzyme-consuming.

## DRUG INTERACTIONS DUE TO INHIBITION EFFECTS

Interaction studies have the following as goals:

- To determine the mechanism of interaction
- To determine the extent of interaction
- To extrapolate the results to the *in vivo* situation and to determine their clinical significance

These aspects can be better approached once the involved enzyme has been identified (see above). A careful kinetic study is the normal way to get all this information, providing a correctly adapted protocol is used. It must, however, be remembered that as most of the concerned enzymes are within membranes, true Km or Ki cannot be obtained; only "apparent" Km, Vm or Ki can be determined.

Depending on the incubation conditions, it is possible to discriminate between:

1. Competitive inhibition, uncompetitive, and noncompetitive inhibition, also called reversible inhibition.
2. Metabolism-dependent inhibition and quasi-irreversible or irreversible inhibitions. For CYP-catalysed reactions they occur only after the oxygen transfer step. In some cases they result in a covalent binding of the substrate to the active site of the enzyme, a case for the so-called suicide substrates.
3. Inhibitions due to reduction of the steady-state concentration of an enzyme.
4. Activation of an enzyme.

1. It is strongly recommended, at least when CYP3A4 is concerned, to use multiple probe substrates to be able to establish a ranking of the different possible substrates or competitors, in order to assess more precisely the mediated drug interactions[14,32]. In addition, this last enzyme seems to be subject to stimulation of activity. Its active site is able to bind more than one substrate, as well as an effector at the same time[33,34]. This situation leads to complex non Michealis-Menten kinetics[35].

2. Some inhibitors first bind to the enzyme active site as a substrate and are then catalytically activated into a reactive species able to bind irreversibly or covalently to an amino acid of the active site, or even directly to the heme. The consequence is a direct altering of the enzyme, which is removed permanently from the catalytic pool[36]. This occurs specifically with terminal acetylenes, macrolides, parathion, some triazoles, isothiocyanates, and substituted hydrazines.

3. Some products may reduce the steady-state concentration of liver P450 isozymes. Fluoxetine, clarithromycin, and the primary metabolite of diltiazem may reduce the CYP3A4 concentration to 72, 39, or 21%, respectively, of its initial value[17] and thereby affect the clearance of compounds eliminated by this route. Macrolide antibiotics like troleandomycin and erythromycin exert their inhibitory action by a similar mechanism[36]. To demonstrate this mechanism, it is necessary to first preincubate the enzyme in the presence of the inhibitor and the cofactors before adding the substrate to start the real incubation, in order to allow the inhibition reaction to occur. *In vivo*, the inhibition effect will largely depend on the time course of administration of the different considered drugs.

4. At least for CYP3A4, homotropic (substrate stimulation) and heterotropic (stimulation by effectors) cooperativity may occur for some substances[34]. This is precisely the case for increased clearance of diclofenac by quinidine[33]. Allosteric effects have been reported for the metabolism of diazepam[37]. Cooperativity in oxidations has been reported for 7,8-benzoflavone[38].

### Extrapolation

Experiments performed on recombinant CYP2C9 with several sulfonamides have shown that the Ki determined on this recombinant enzyme is very similar to that found on human liver microsomes[30]. Moreover, the increase of tolbutamide AUC in humans, predicted from these *in vitro* studies, agreed well with clinical observations[30].

"Blind" assessment of CYP-associated metabolism and interactions in *in vitro* systems[39] has shown that, for some substances, the predictions made from *in vitro* studies are successfully extrapolated to the *in vivo* situation. Reliability of the prediction of human hepatic clearance from *in vitro* studies on human liver microsomes depends largely on the extrapolation parameters used. In some circumstances, incorporation of information from *in vivo* animal studies may provide more reliable predictions[40].

Classical Michaelis-Menten kinetics are based on simple equations assuming one single binding site per enzyme, infinite dilution, and one single enzyme — often unverified assumptions, which may not be applicable to CYP enzymes that are deeply imbedded in a lipid membrane. In particular, CYP3A4, the most important hepatic CYP, is known to exhibit unusual kinetic properties[41,42]. Its active site seems able to bind simultaneously more than one substrate or a combination of substrate, activator, and/or inhibitor. Resulting kinetics are not easily interpreted in terms of drug interaction[43,45].

## DRUG INTERACTIONS DUE TO INDUCTION EFFECTS

Several drugs are able to induce drug metabolism: cigarettes, alcohol, rifampicine, some antibiotics, phenobarbital, dexamethasone, some glucocorticoïds, etc. (see http://medicine.iupui.edu/flockhart/index.html). Smoking is associated with increased clearance of psychotropic medications, such as tiotixene, fluphenazine, haloperidol, and olanzapine, and also of some benzodiazepines like diazepam, lorazepam, or alprazolam[44]. Docetaxel and paclitaxel are able to induce CYP3A4 in cultured human hepatocytes, leading to 2.2- and 5-fold increases, respectively, in testosterone-6β-hydroxylase activity, the common substrate of this enzyme[46].

When potential inducers are used for long-term therapy, the clearance of other drugs can be seriously modified. Hyperforin, a component of St. John's wort (*Hypericum perforatum*), an herbal remedy used in the treatment of depression, is a strong inducer of CY3A4, acting on the pregnane X receptor (PXR) that serves as a key regulator of transcription of the mRNA coding for CYP3A4[47,48]. This product increases the metabolism of indinavir, cyclosporine, contraceptives, and other CYP3A4-metabolised drugs[12]. Some HIV protease inhibitors induce both intestinal P-glycoprotein and hepatic CYP3A4 and may consequently reduce drug exposure after multiple doses[62].

Induction of drug-metabolising enzyme activity is a complex phenomenon whose mechanism is not yet elucidated. Induction requires translation of a (or several) gene(s) to increase the biosynthesis of the corresponding protein. This involves binding of an inducer to a receptor and activation of the receptor or a regulator, a product- and concentration-dependent process. In addition, the induction can be modulated by other regulators or receptors, namely glucocorticoid receptors[49,50]. Therefore, prediction of induction and its extent remains a hazardous task.

In some circumstances CYP3A4 induction can be evaluated *in vivo*, in humans, using either the erythromycin breath test developed by Watkins[50] or by measuring the urinary excretion of 6β-hydroxycortisol[51,52].

## *IN SILICO* PREDICTION OF DRUG INTERACTIONS

The decisive progress yielded nowadays by ongoing research, along with the major new possibilities offered by computer science, will most probably allow rapid development of reliable "*in silico*" approaches to predict DDI.

SAR and QSAR studies, elucidation of the three-dimensional structure of proteins, receptors, enzymes, etc., will soon allow an objective representation of the binding of a drug to its biochemical target. This will allow a rapid comparison of different products and the determination of the critical chemical functions, structures, and affinities involved in an enzymatic process. A better knowledge of the kinetic parameters and their relation to the biochemical or pharmacological process is a necessary step toward this goal[53]. Research going on in different laboratories will certainly contribute very efficiently into this direction[54,55].

Large databases are becoming available, namely the numerous results issued from HTS (high throughput screening), that allow correlation studies between *in vitro* and *in vivo* data. New software applications are being developed to handle these data and to extrapolate the results to similar situations or products.

Recently, a dynamic computer-based method, called Quantitative Drug Interactions Prediction System (Q-DIPS), has been developed to make both qualitative deductions and quantitative predictions on the basis of databases containing updated information on CYP substrates, inhibitors, inducers, and pharmacokinetic parameters[56,57]. Three-dimensional structure-activity relationship studies are improving very rapidly, as is our knowledge of the architecture of the enzyme active site. This, combined with computer modelling, will allow a successful design of pharmacophore models of great help for the prediction of possible DDI and their mechanism[58].

## DISCUSSION

Is it possible to answer clearly the question: "Are DDI predictable?"

The tools in our hands today certainly allow us to study the phenomenon correctly and, in a large number of cases, to predict correctly, at least on a qualitative basis, DDI from simple *in vitro* studies. Moreover, as recommended by the regulatory agencies (FDA, EMEA), this should be done for all new drugs under development. From a financial and economical point of view, an early detection of possible DDI is certainly an important issue, since it offers the possibility to reject rapidly the NCE or to modify its chemical structure accordingly.

To avoid DDI, it is desirable to develop drugs that are neither potent CYP inducers nor inhibitors, the metabolism of which is not affected by other common drugs. In reality, this is not achievable, and the objective of drug developers is to reduce these events as much as possible by an adequate choice of the NCE. For economical reasons, this choice should occur as early as possible during development and, preferably, during the discovery phase[35].

At present, most basic information on drug metabolism, inhibition, and induction is obtained from *in vitro* experiments. The development of cost-effective techniques during the last decade has certainly increased this phenomenon while improving its efficacy.

Optimising drug development was the subject of a recent conference of the European Federation of Pharmaceutical Sciences (EUFEPS) devoted to strategies to assess drug metabolism and interactions. The consensus report issued from this conference[59] constitutes an excellent review of the state of the art concerning the different methodological aspects.

But, we have to remain cautious and to be aware that we are still unable to predict all the possible interactions (those occurring on not-yet-studied enzymes, poorly understood receptors or signal transducers, etc.). Obtaining negative results never allows us to assume the complete absence of DDI. Unknown processes and inadequate experimental approaches are still possible misleading sources. Getting a positive result does not mean that DDI will automatically occur *in vivo*. The results have to be balanced as a function of the extrapolation parameters taken into account and the relative contribution of the concerned metabolic pathway to the overall clearance of the drug. For instance, the rate of metabolism and the possible interaction depend largely on the local concentration of enzyme, substrate, and inhibitor. Usually, these concentrations are not precisely known.

Extrapolation of *in vitro*–measured data to an *in vivo* situation remains a hazardous task, depending largely on the parameters used for this purpose[40].

Although *in vitro* experiments give rapid, very accurate, and useful results, their extrapolation to the clinical situation is fraught with difficulties. No single quantitative model representing all the different facets of such a complex task as predicting and evaluating the clinical risk of DDI can be sufficiently simple and comprehensive to become widely useful and usable. The constantly increasing number of parameters to take into consideration makes the models more and more complex and less applicable on a large scale[57]. Unless a breakthrough is achieved in computer modelling of the 3D structure of the enzyme, and particularly of its active site, the predictivity of the *in vitro* findings will remain essentially limited to homologous series of products. At any rate, extrapolation will always have to be performed very cautiously and still needs the accumulation of large amounts of data to be validated, even for limited applications.

A global approach to drug interaction problems necessitates rational interaction, investigations, associating sequentially *in vitro* studies, *in vitro/in vivo* extrapolation, and finally, relevant and well-designed *in vivo* clinical studies[57].

## ACKNOWLEDGEMENTS

I am grateful to all my colleagues from the European concerted action COST B15, entitled "Modelling in Drug Development" for numerous stimulating discussions. I would like to express my thanks to Prof. A.M. Batt and Dr. L. Ferrari (Centre du médicament, Université de Nancy, France) for a critical reading of the manuscript.
